# High Visceral to Subcutaneous Fat Ratio Is Associated with Increased Postoperative Inflammatory Response after Colorectal Resection in Inflammatory Bowel Disease

**DOI:** 10.1155/2018/6270514

**Published:** 2018-04-03

**Authors:** Yao Wei, Feng Zhu, Jianfeng Gong, Jianbo Yang, Tenghui Zhang, Lili Gu, Weiming Zhu, Zhen Guo, Yi Li, Ning Li, Jieshou Li

**Affiliations:** ^1^Department of Emergency and Critical Care Medicine, The First Affiliated Hospital of Soochow University, Pinghai Road 900, Suzhou 215031, China; ^2^Department of General Surgery, Jinling Hospital, Medical School of Nanjing University, East Zhongshan Road 305, Nanjing 210002, China

## Abstract

**Aim:**

Excessive postoperative inflammatory response, which is characterized by overproduction of cytokines, often leads to complications after colorectal surgery. However, the impact of body composition on postoperative inflammatory response is largely unknown. The aim of this study is to elucidate whether body fat amount and its distribution affects postoperative inflammation after colorectal surgery in IBD patients.

**Methods:**

Eighty-six patients undergoing colorectal resection for IBD from June 2014 to Jan 2017 were enrolled. Abdominal CT images within one week prior to surgery were assessed for visceral fat, subcutaneous fat, and muscle mass. Postoperative inflammatory response was evaluated using serum CRP, PCT, and IL-6 levels on postoperative days 1, 3, and 5. Univariate analysis was conducted to identify risk factors for infectious complications. The correlation between body composition and postoperative plasma concentration of inflammatory markers was analyzed using a linear regression model. ROC curve was applied to analyze the effect of different body composition parameters on postoperative infectious complications and to determine the relationship between inflammatory markers and infectious complications.

**Results:**

Neither volume of fat or muscle was related to postoperative plasma concentrations of CRP, IL-6, and PCT. However, visceral to subcutaneous fat ratio was associated with PCT levels on postoperative days (POD) 1, 3, and 5, with the highest regression coefficient on POD1 (*β* = 0.360; 95% CI, 0.089–0.631; *P* = 0.010). Body composition did not predict postoperative infectious complications, while CRP on POD 3 was predictive of infectious complications.

**Conclusion:**

Increased visceral to subcutaneous fat ratio was associated with postoperative inflammatory response in IBD patients undergoing colorectal resection. This may partly explain the increased incidence of postoperative complications in patients with visceral obesity.

## 1. Introduction

Elevated postoperative inflammatory response is common after colorectal resection, increasing morbidity by causing postoperative infectious complications and prolonging length of stay. Different measures have been proposed to ameliorate this situation. For example, preoperative administration of dexamethasone has been proved to reduce postoperative inflammatory response after surgery for colorectal cancer [1]. A phase II randomized study found that postoperative ghrelin administration is effective in inhibiting inflammatory markers and postoperative pulmonary complications after esophagostomy [2]. Another study comparing different surgical and analgesic techniques showed that open surgery has a greater impact on postoperative inflammatory response than laparoscopic surgery, while no significant difference was detected between epidural and intravenous analgesia [3].

The relationship between body composition and postoperative inflammatory response has drawn much attention recently. Generally, the human body consists of fat-free mass (FFM), visceral fat mass (VFM), and subcutaneous fat mass (SFM). Reisinger et al. [4] reported that sarcopenia, characterized by loss of skeletal muscle mass, is associated with increased postoperative inflammatory response after colorectal surgery. However, the impact of body fat composition on surgical outcome is largely unknown.

Visceral adipose tissue, largely distributed in the abdominal cavity, shows high hormonal and metabolic activities via secretion of various proinflammatory cytokines such as TNF-*α* and IL-6. Previous study showed that abdominally obese patients with excess visceral adipose tissue have elevated IL-6 and TNF-*α* levels and reduced adiponectin levels [5, 6]. In the case of IBD patients, Peyrin-Biroulet et al. [7] proved that mesenteric fat was an extrahepatic source of C-reactive protein (CRP) in Crohn's disease (CD). Recent studies also found that fat subtype and distribution can predict postoperative infectious complications after bowel resection for CD, and visceral fat is associated with high risk of postoperative recurrence in CD [8, 9]. Similarly, another study also found excessive visceral fat to be an independent risk factor for both pancreas-related infection and anastomotic leak following gastrostomy [10].

Based on the aforementioned facts, we proposed a hypothesis that patients with different adipose amounts and compositions may have varied postoperative inflammatory responses. Therefore, the aim of this study was to find out if body fat composition is associated with postoperative inflammatory response in IBD patients.

## 2. Methods

### 2.1. Study Subjects

This was a retrospective study approved by the Ethical Committee of Jinling Hospital. Consecutive patients with IBD who underwent colorectal resection at Jingling Hospital from June 2014 to Jan 2017 were included in this study. Subject selection criteria included (1) patients aged 18–65 years, (2) patients undergoing colonic or rectal resection for Crohn's disease or ulcerative colitis, and (3) availability of a CT scan of the abdomen within 7 days prior to abdominal surgery. Exclusion criteria were as follows: (1) previous history of abdominal surgery; (2) unavailable inflammatory markers on postoperative days (POD) 1, 3, and 5; and (3) preoperative systematic inflammatory response syndrome (SIRS), manifested by two or more of the following conditions:

- Temperature > 38°C or <36°C
- Heart rate > 90 beats per minute
- Respiratory rate > 20 breaths per minute or PaCO_2_ < 32 mmHg
- White blood cell count > 12,000/cu mm, <4000/cu mm, or >10% immature (band) forms [11]

### 2.2. Assessment of Body Fat Composition

CT data were obtained from the PACS (Picture Archiving and Communication Systems) of the patients. CT images obtained within 7 days before surgery represent the amount of muscle and subtype of body fat status at the time of surgery. A single cross-sectional CT image of a 3 mm thick slice at the level of L3 was obtained with the subject at supine position. Muscle, subcutaneous fat, and visceral fat were measured in square centimeters on the basis of the pixel count as previously described [12]. Briefly, the “Magic Wand” tool within Photoshop (Adobe Systems, San Jose, CA) was used to outline and measure the muscle, subcutaneous fat, and visceral fat in each stored digital image [13, 14].

### 2.3. Measurement of Clinical Parameters

Blood samples were collected regularly on the morning preoperatively and on POD1, POD3, and POD5. CRP, IL-6, alb, and PCT concentrations were measured by the laboratory department and collected from the database. Demographic information such as gender, age, and body mass index (BMI) were retrieved from medical records. Intraoperative data including operative site, operation time, and estimated blood loss were collected in operation charts. Preoperative use of 5-ASA and steroids was defined as 4 weeks before surgery, and preoperative use of immunomodulators or biologics (azathioprine, 6-mercaptopurine, or infliximab administered) was defined as 8 weeks before surgery. Patients' history was reviewed for diagnosis, postoperative length of stay, and complications. Postoperative infectious complications were classified into surgical site infection (SSI) and remote site infection (RSI) [15–18]. SSI can be further divided into incisional (wound infection) and others (anastomotic leak and intra-abdominal collection). RSI includes urinary tract infection, septicemia, antibiotic enterocolitis, and central line infection [19]. But *Clostridium difficile* colitis and pneumonia were not included in postoperative infectious complications.

### 2.4. Statistics

Data for demographic characteristics are expressed as mean ± SD or median (range), depending on whether it is normally distributed according to the Levene test. Univariate analysis was conducted to identify any risk factors of postoperative infectious complications. Any factors with a *P* < 0.10 were further assembled for logistic regression analysis after validating the absence of multicollinearity. To evaluate whether a different body composition is associated with inflammatory response after colorectal surgery, linear regression models were applied after testing the sample for linearity, independence, normality, and equal variance. Different body composition parameters such as the skeletal muscle area (SMA), visceral fat area (VFA), subcutaneous fat area (SFA), and ratio of VFA/SFA were screened. The regression coefficient *β* was calculated, and a *P* value of <0.05 was considered as statistically significant. ROC curves were drawn to analyze the effect of different body composition parameters and inflammatory markers on postoperative infectious complications. All analyses were conducted using SPSS version 20.0 (SPSS Inc., an IBM company, Chicago, IL).

## 3. Results

### 3.1. Patients' Characteristics

**Figure 1:**
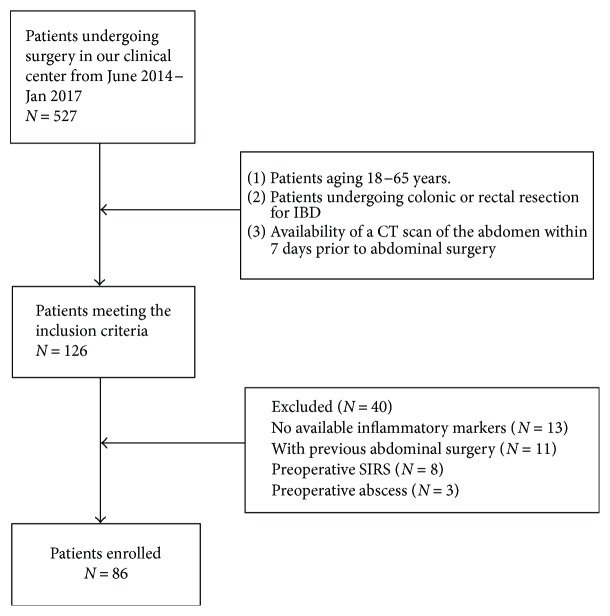
Flow chart demonstrating the selection process of the patients.

A total of 527 IBD patients underwent colorectal surgery in our center from June 2014 to Jan 2017. 126 patients met the inclusion criteria. 40 patients were excluded subsequently (18 without accessible results of inflammatory markers, 11 with previous abdominal surgery, 8 with preoperative SIRS, and 3 with preoperative abdominal abscess). The selection process was demonstrated in Figure 1. Finally, 86 patients were enrolled in our study. General characteristics and perioperative parameters of patients were listed in Table 1. 51 (59.3%) patients were male. The average age was 36.2 (14.0). The mean BMI was 18.2 ± 2.83 kg/m^2^. The mean VFA values in males and females were 62.30 ± 39.0 cm^2^ and 74.10 ± 31.80 cm^2^, respectively (*P* = 0.142). The mean SFA values in males and females were 77.0 ± 52.40 cm^2^ and 141.80 ± 69.90 cm^2^, respectively (*P* < 0.001). The ratio of VFA to SFA was defined as mesenteric fat index (MFI), and males had higher MFI compared to females (0.74 ± 0.33 versus 0.51 ± 0.23, *P* < 0.001).

Among these 86 patients, 64 (74.4%) patients were diagnosed with Crohn's disease, and 22 (25.6%) patients were diagnosed with ulcerative colitis. 42 (48.8%) patients underwent ileocecal/right colectomy, 4 (4.65%) patients underwent transverse colectomy, 10 (11.6%) patients underwent left colectomy, 8 (9.30%) patients underwent sigmoid/rectal resection, and 22 (25.6%) patients underwent total colectomy. Besides, 28 (32.6%) patients underwent laparoscopic surgery, and 58 (77.4%) patients underwent open surgery. Estimated intraoperative blood loss was 88.1 (10–300) mL. Operation time was 195.6 ± 64.0 min. Postoperative length of stay was 10.7 (4–44) day.

### 3.2. Postoperative Inflammatory Response

**Figure 2:**
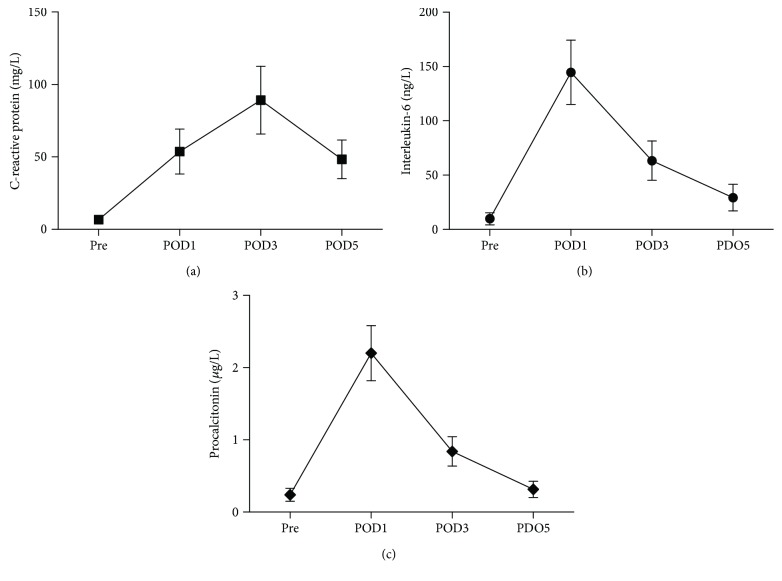
Changes over postoperative time in C-reactive protein (a), interleukin-6 (b), and procalcitonin (c). All of the three plasma inflammatory markers increased greatly after the surgery took place. CRP had a peak on POD 3, while IL-6 and PCT decreased from POD 1 to 5. Pre: preoperative; POD *x*: postoperative day *x*.

Postoperative inflammatory markers were shown in Figure 2. Generally, preoperative inflammatory markers are comparatively low (CRP 6.63 ± 1.94 mg/L, IL-6 9.86 ± 4.25 ng/L, and PCT 0.09 ± 0.28 *μ*g/L). Both postoperative IL-6 and PCT showed a descending trajectory from POD 1 (IL-6147.02 ± 47.8 ng/L and PCT 2.30 ± 1.10 *μ*g/L) to POD 5 (IL-6 33.87 ± 14.01 ng/L and PCT 0.30 ± 0.16 g/L), while CRP peaked on POD 3 (91.05 ± 29.80 mg/L).

Linear regression analysis showed that MFI had a positive correlation with PCT from POD 1 to 5, with the highest coefficient appearing on POD 3 (*β* = 0.360; 95% CI, 0.089–0.631; *P* = 0.010). Regression coefficients *β* and confidence intervals are summarized in Table 2. However, BMI, SMA, SFA, and VFA alone were not related to postoperative inflammatory markers, and neither CRP nor IL-6 was correlated with MFI.

### 3.3. Postoperative Infectious Complications

**Figure 3:**
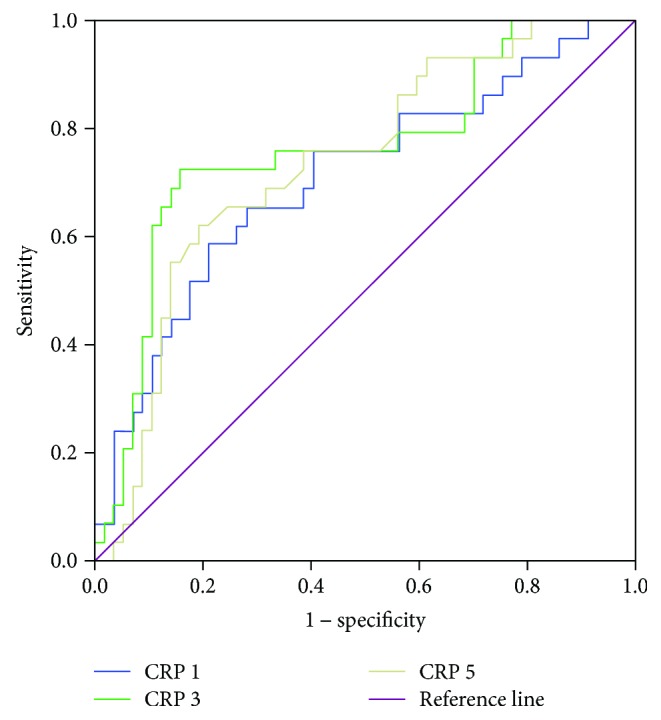
Diagnostic accuracy of inflammatory markers with regard to development of postoperative infectious complications. The AUC values were 0.708 (*P* = 0.002), 0.760 (*P* < 0.001), and 0.734 (*P* < 0.001) for CRP on postoperative days (POD) 1, 3, and 5, respectively; Youden index revealed that a CRP level of >55.3 mg/L on POD 3 is predictive of postoperative complications with the sensitivity being 62.1% and the specificity being 73.7%.

Of the 86 patients, 11 had Clavien-Dindo I complications (6 with ileus and 5 with wound infection not requiring intravenous antibiotics), 22 had Clavien-Dindo II complications (14 with wound infection requiring intravenous antibiotics, 5 with pouchitis, and 3 with intra-abdominal infection), and 2 had Clavien-Dindo III complications (1 with intra-abdominal infection requiring stoma reconstruction under general anesthesia and with 1 intra-abdominal infection requiring reoperation on POD 10). A total of 29 (33.7%) patients had postoperative infectious complications (wound infection in five class I patients and in all the class II and III patients). Univariate analysis showed that preoperative albumin level, steroid use, diagnosis, surgical site, and operation time were associated with postoperative infectious complications.  Table 3 shows that preoperative albumin level (*P* < 0.001, *β* = −0.135, OR 0.873, 95% CI 0.834–0.914), steroid use (*P* = 0.038, *β* = 0.982, OR 2.671, 95% CI 0.928–7.683), and operation time (*P* = 0.022, *β* = 0.010, OR 1.010, 95% CI 1.001–1.019) were associated with postoperative infectious complications. BMI of patients with and without infectious complication after surgery was 18.6 kg/m^2^ versus 17.9 kg/m^2^ (*P* = 0.314). Likewise, none of the body composition parameters, including BMI, MFI, VFA, and SFA, were associated with postoperative infectious complications. Furthermore, the relationship between CRP levels and the development of postoperative infectious complications in IBD patients were plotted in ROC curves (Figure 3). It demonstrated that the CRP levels on POD 3 were predictive of postoperative infectious complications, with an AUC value of 0.760 (95% CI: 0.646–0.875, *P* < 0.001). The optimal cutoff value was 55.3 mg/L with the sensitivity being 62.1% and the specificity being 73.7%.

## 4. Discussion

Surgery leads to predictable inflammatory response afterwards, causing acute phase responses initialized by production of proinflammatory cytokines including IL-1, IL-6, IL-8, and TNF-*α*. Such cytokines act to mobilize the innate immune system, resulting in the activation of neutrophils, macrophages, and platelets [20]. Besides, the presence of systemic inflammatory response can suppress cytotoxic immunity and may promote the development of postoperative complications [21]. Of all the commonly examined inflammatory markers, CRP and IL-6 are both useful markers representing the magnitude of postoperative inflammation [22]. Also, PCT is another effective marker of postoperative inflammatory response showing higher specificity than CRP in differentiating between inflammation and infection according to [23].

Previous articles showed variations in fat volume, and distribution may impact surgical outcomes [10, 24–28]. However, neither visceral fat mass nor subcutaneous fat mass alone can capture changes in fat distribution and overall body composition. As a result, our study found no significant correlation between them and postoperative inflammatory response.

On the other hand, visceral obesity measured by visceral fat area (VFA) and VFA/SFA ratio has been examined as a predictor of colorectal surgery outcomes [12, 28, 29]. The negative effects of visceral adipose tissue are thought to be mediated by the state of chronic inflammation associated with cytokines such as TNF-*α*, IL-6, and IL-8 [13].

In our study, we found that MFI reflects the composition of body fat, and an increased MFI is related to higher PCT after operations from POD1 to POD5. That is to say, the ratio of visceral fat area/subcutaneous fat area can be a predictor of inflammatory response to surgery. Surgeons can, in advance, adopt measures such as physical exercise to modify body composition to prevent severe inflammatory response after surgery. However, we found that muscle mass is not associated with increased postoperative inflammatory response, which is inconsistent with the conclusion of other studies [4, 30]. This might be caused by the insufficient sample size of our study. Besides, Stidham et al. [8] found that subcutaneous to visceral fat ratio was predictive of infectious complications, and Ding et al. [31] described the relationship between higher visceral fat and postoperative complications in Crohn's disease. In contrast, our study found no difference in body composition between patients with infectious complications and those without, which might be caused by the inclusion of ulcerative colitis patients in our cohort and the different technique of body composition analysis applied. These conflicting findings together warrants further prospective, multicenter studies with larger sample size to elucidate the relationship between body fat and postoperative infectious complications.

Apart from MFI and other body composition parameters, we also evaluated the inflammatory biomarkers' capacity to predict infectious complications. Mokart et al. [32] reported that serum IL-6 concentration is higher in patients undergoing a range of abdominal surgeries who develop postoperative SIRS or sepsis than in those with uneventful recovery. Raised IL-6 on postoperative day 1 predicts postoperative sepsis in patients undergoing major gastrointestinal surgery. In our study, 29 (33.7%) patients had infectious complications after surgery. ROC analysis showed that neither IL-6 nor PCT was predictive of postoperative infectious complications. This is different from the previous results and warrants more studies. A CRP level of >170 mg/L measured on POD 3 is another effective predictor of infectious complications in patients with colorectal cancer according to Platt et al. [19]. Our results in IBD patients agree with the previous study with the optimal threshold of CRP > 55.3 mg/L on POD 3 in predicting postoperative infectious complications.

Several limitations must be considered when interpreting the results of our work. First, decisions on surgical timing, preoperative optimization strategies, and surgical methods and the surgeons were not standardized among patients, introducing selection bias into the results. Second, measures such as propensity-score matching were not introduced in our study to eliminate the potential confounding variables. Third, the sample size is small and comes from a single center. Therefore, a multicenter study with a larger sample size is required to validate our findings.

Despite the limitations of a retrospective study design, these data indicate that a more detailed fat assessment may aid in the prediction of postoperative inflammatory response and infectious complications in general surgery.

## Figures and Tables

**Table 1:** Basic characteristics of the patients (*n* = 86).

	With infectious complications (*n* = 29)	Without infectious complications (*n* = 57)	*P*
Age	36.7 (13.3)	35.9 (12.9)	0.628^a^
Sex (male : female)	18 : 11	33 : 24	0.710^b^
Muscle cross section area	136.1 (35.4)	132.3 (42.5)	0.679^a^
Subcutaneous fat area (cm^2^)	106.1 (68.9)	102.0 (67.7)	0.793^a^
Visceral fat area (cm^2^)	67.7 (34.7)	66.7 (37.7)	0.904^a^
Mesenteric fat index (MFI)	2.18 (2.49)	1.98 (1.91)	0.683^a^
BMI (kg/m^2^)	18.6 (2.6)	17.9 (2.9)	0.314^a^
Preoperative albumin (g/L)^†^	34.8	38.2	<0.001^a^
Preoperative medication			
5-ASA	14	23	0.483^b^
Immunomodulators	14	18	0.130^b^
Glucocorticoids	11	10	0.037^b^
Biologics	1	4	0.659^b^
Diagnosis			
Crohn's disease	20	44	—
Ulcerative colitis	12	10	0.001^b^
Surgical site			
Ileocecal/right colectomy	13	29	—
Transverse colectomy	1	3	—
Left colectomy	4	6	—
Sigmoid/rectal resection	2	6	—
Total colectomy	12	10	0.001^c^
Surgical technique (laparoscopic : open surgery)	11 : 18	17 : 40	0.448^b^
Estimated blood loss (mL), median (range)	101.3 (10–300)	101.3 (10–300)	0.164^d^
Operative time (min), mean (SD)	182.6 (58.2)	221.0 (68.0)	0.008^a^
Length of stay (day), median (range)	14.3 (7–44)	8.8 (4–22)	<0.001^d^
Postoperative complications (Clavien-Dindo classification)			
I	11	—	—
II	22	—	—
III	2	—	—
IV or above	0	—	—

Data presented as mean (SD) or median (range). Data were calculated using ^a^Student's *t*-test, ^b^*χ*^2^ test, ^c^Fisher's exact test, and ^d^Mann–Whitney *U* test. ^†^Preoperative albumin level was not recorded for 4 patients.

**Table 2:** Regression of body composition and PCT on different postoperative days.

	*β*	95% CI		*P*
POD 1				
MFI	0.360	0.089	0.631	0.010
Muscle area	−0.001	−0.018	0.016	0.938
Visceral fat area	0.017	0.006	0.032	0.175
Subcutaneous fat area	−0.013	−0.022	−0.004	0.118
POD 3				
MFI	0.131	0.023	0.239	0.018
Muscle area	−0.001	−0.007	0.006	0.772
Visceral fat area	0.010	0.004	0.019	0.192
Subcutaneous fat area	−0.005	−0.009	−0.002	0.213
POD 5				
MFI	0.130	0.062	0.198	<0.001
Muscle area	0.001	−0.002	0.003	0.644
Visceral fat area	0.003	0.001	0.007	0.421
Subcutaneous fat area	−0.002	−0.003	<0.001	0.271

POD *x*: postoperative day *x*; CI: confidence interval.

**Table 3:** Logistic regression of variables associated with postoperative infectious complications.

Factors	*β*	Odds ratio	95% CI	*P*
Albumin level	−0.135	0.873	0.834–0.914	<0.001
Steroid use	0.982	2.671	0.928–7.683	0.038
Total colectomy	0.229	1.257	0.370–4.274	0.714
Operation time	0.010	1.010	1.001–1.019	0.022

## Additional Points

*Statement: What Does This Paper Add to the Literature?* A part of IBD patients with normal body weight could suffer from serious inflammatory response after colorectal surgery. This paper found that the ratio of visceral to subcutaneous fat affects postoperative inflammatory response in an IBD patient, and this may aid in the prediction of postoperative inflammatory response and infectious complications in general surgery.

## Abbreviations

BMI: Body mass index
CD: Crohn's disease
CI: Confidence interval
CT: Computed tomography
IBD: Inflammatory bowel disease
LOS: Length of stay
MFI: Mesenteric fat index
SFA: Subcutaneous fat area
VFA: Visceral fat area
SIRS: Systemic inflammatory response syndrome
SSI: Surgical site infection
RSI: Remote site infection
CRP: C-reactive protein
IL-6: Interleukin-6
PCT: Procalcitonin
5-ASA: 5-Aminosalicylic acid
PACS: Picture Archiving and Communication Systems.
